# A high urinary urobilinogen/serum total bilirubin ratio indicates acute hepatic porphyria in patients with abdominal pain

**DOI:** 10.1038/s41598-023-48824-9

**Published:** 2023-12-04

**Authors:** Chengyuan Song, Yuan Liu

**Affiliations:** 1 Department of Neurology, Qilu Hospital of Shandong University, Jinan, 250012 People’s Republic of China; 2 Department of Endocrinology, Qilu Hospital of Shandong University, #107 of Culture Road, Jinan, 250012 Shandong People’s Republic of China; 3 Institute of Endocrine and Metabolic Diseases of Shandong University, Jinan, 250012 People’s Republic of China

**Keywords:** Pain, Gastrointestinal diseases, Biological techniques, Endocrinology, Gastroenterology

## Abstract

Acute hepatic porphyria (AHP) has always been a diagnostic dilemma for physicians due to its variable symptoms. Correct diagnosis mainly depends on the detection of an elevated urinary porphobilinogen (PBG), which is not a routine test and highly relies on the physician’s awareness of AHP. In the present study, we identified a more convenient indicator during routine examinations to improve the diagnosis of AHP. We found that AHP patients showed a significant higher “FALSE” urinary urobilinogen level caused by urinary PBG during the urinalysis when detected by strips impregnated with Ehrlich reagent (*P* < 0.05). And a remarkable increase in the urinary urobilinogen/serum total bilirubin ratio was observed in AHP patients. The area under the ROC curve of this ratio for AHP was 1.000 (95% confidence interval 1.000–1.000, *P* < 0.01). A cutoff value of 3.22 for this ratio yielded a sensitivity of 100% and a specificity of 100% to distinguish AHP patients from the controls. Thus, we proved that a “falsely” high urinary urobilinogen level that was adjusted by the serum total bilirubin level (urinary urobilinogen/serum total bilirubin ratio) could be used as a sensitive and specific screening marker for AHP in patients with abdominal pain.

## Introduction

Acute hepatic porphyria (AHP) is a rare but life-threatening disease. This autosomal dominant genetic disorder was caused by the defect of the enzymes during heme biosynthesis pathway^1^. Due to the partial deficiency of these enzymes, excess amounts of 5-aminolevulinic acid (ALA) and porphobilinogen (PBG) accumulate and cause dysfunction of the autonomic system and neuropathy^2^. During the acute attack of AHP, abdominal pain is the most common presentation and the leading reason for the emergency visit^3^. However, due to the various symptoms and nonspecific routine laboratory test results, the diagnosis of AHP has always been a significant challenge for the physicians, and a delay in diagnosis or even misdiagnosis is very common^3–5^. There are four classes of AHP: acute intermittent porphyria (AIP), hereditary coproporphyria (HCP), variegate porphyria (VP), and aminolevulinic acid dehydratase deficient porphyria (ADP)^6^. Since ADP is extremely rare and presenting a different laboratory results^7^, we mainly focused on AIP, HCP and VP, which are more common subtypes with elevated urinary PBG during attack, in the present study.

The examination of urinary PBG is the key test in patients with suspected AIP, HCP and VP^8^. However, as an unconventional examination, the urinary PBG is detected only when the physicians suspect the possibility of AHP. Unfortunately, the awareness of this rare disease in developing countries is really poor^3,9,10^. Moreover, only several hospitals in mainland of China carried out the detection of urinary PBG, not to mention the quantitative ALA, urinary and plasma porphyrin levels^11^. Gene test is an alternative way; however, it is money-consuming and time-consuming. These all greatly compromise the diagnosis of AHP. Therefore, it is of great importance to identify a more convenient indicator during routine examinations to improve the diagnosis of AHP.

## Methods

### Study design and participants

We reviewed patients with AHP who were admitted to Qilu Hospital, Shandong University between 2015 and 2022. Patients were diagnosed as urinary PBG-elevated AHP if they met the following diagnostic criteria^11^, (i) acute attack symptoms: severe abdominal pain in the absence of significant abdominal tenderness and neuropathic symptoms such as seizures; and (ii) a positive result for urinary PBG. Among the AHP, the AIP (OMIM 176000) is the most common type which is an autosomal dominant disorder caused by mutation in hydroxymethylbilane synthase (HMBS) gene leading to half-normal HMBS enzymatic activity^12^.Since the detection of urinary PBG is not widely carried out in China, so a genotype with a mutation in the HMBS gene also considered as a diagnostic criteria.

According to studies of AHP in China, the common misdiagnosis of AHP includes intestinal obstruction, pancreatitis, appendicitis, cholecystitis, and gallstones^13^. We randomly enrolled 100 patients with intestinal obstruction(N = 25), pancreatitis(N = 25), appendicitis(N = 25), and gallbladder diseases(N = 25) as the control groups from the emergency department between January to August of 2022. The urinary PBG were tested in the control groups to exclude AHP. Patients with a history of tumor, hemolytic disease, liver cirrhosis, splenomegaly, and autoimmune hemolytic anemia were excluded.

The study protocol was approved by the Medical Ethics Committee of Qilu Hospital, Shandong University, China (approval number: KL2021027). Informed consent was obtained from all the patient who participated in this study. The study was conducted in accordance with the principles of the Declaration of Helsinki.

### Data collection

The medical history, physical examination, as well as blood and urinary biochemical results were collected from the Electronic Medical Records of the patients. The blood biochemical tests included hemoglobin, alanine transaminase, aspartate transaminase, total bilirubin, conjugated bilirubin, unconjugated bilirubin, amylase, serum sodium. These tests were performed using standard hospital laboratory techniques. BC-5390CRP (Mindray Biomedical Electronics Co., Ltd, China) was used for the hemoglobin examinations. The remaining serum markers were measured using a Vitros 5600 (Ortho Clinical Diagnostics, USA).

### Urine urobilinogen test

Urinary urobilinogen was carried out using Ehrlich reaction on urine test strips (CombiScreenR11SYS PLUS). The strip can also be used for detection of urinary bilirubin, ketone, ascorbic acid, glucose, protein, blood, pH, nitrite, leukocytes and specific gravity. The strips were inserted in a fresh random urine sample for one second and then the results were compared with the color scale for urobilinogen after 30–60 s. There are five grades of a pink colored scale increasing in intensity with increasing of urobilinogen level. The first color grade means normal, second 35 umol/l, third 70 umol/l, fourth 140 umol/l and fifth 200 umol/l. Qualitative detection of urinary PBG was carried out using Watson–Schwartz test and the Ehrlich's reagent was purchased from Solarbio life sciences, China.

### Watson–Schwartz test

The Watson–Schwartz test has been a widely used method for urinary PBG detection for more than 80 years. Ehrlich's reagent in the first step reacts with PBG and forms a red condensation product. However, PBG is not the only substance that can react with Ehrlich's reagent. Ehrlich's reagent also reacts with urobilinogen^10^. As such, in the second step of Watson–Schwartz test, chloroform is added to the solution to distinguish the PBG-Ehrlich compound from the urobilinogen-Ehrlich complex. Red color in the aqueous phase of the test tube after adding chloroform illustrates the existence of the PBG-Ehrlich compound while red color in the chloroform phase proves the existence of the urobilinogen-Ehrlich complex.

### Statistical analyses

All the statistical analyses were performed in SPSS (version 24.0, SPSS, Chicago, IL, USA). Normally distributed continuous data (assessed by the Shapiro–Wilk test) are presented as mean ± SD and were analyzed by the independent sample t-test. Non-normally distributed data are presented as median (percentage) and were analyzed by the Mann–Whitney U test. The diagnostic performance was measured as sensitivity, specificity, and accuracy. The cut-off for optimal clinical performance was determined by the receiver operator characteristic (ROC) curve. Results were considered significant at a *P* < 0.05.

### Ethics approval and consent to participate

The study protocol was approved by the Medical Ethics Committee of Qilu Hospital, Shandong University, China (approval number: KL2021027). Informed consent was obtained from all the patient who participated in this study. The study was conducted in accordance with the principles of the Declaration of Helsinki.

## Results

### Demographic and clinical characteristics of the AHP patients

Between 2015 and 2022, 12 AHP patients were admitted into our hospital. The mean age at diagnosis was 27.7 ± 6.8 years old, with a female/male ratio of 11:1. These patients had 4.8 ± 1.9 acute attacks with a maximum of eight attacks before the correct diagnosis. Abdominal pain (100%), pain in the extremities (50.0%), seizures (33.3%), and dark urine (16.7%) were common clinical presentations. During the physical examinations, tachycardia and hypertension were observed in 75.0% and 58.3% of the patients, respectively. None of the patients reported skin lesions. In seven of the patients, AHP was triggered by hormonal variations during pregnancy or the menstrual cycle. In one patient, AHP was triggered by alcohol intake. No clear causes were recorded for the remaining four patients (Table1).

**Table 1:** Clinical profile of the patients with acute hepatic porphyria.

Case	Sex	Age	Precipitating factors	Abdominal pain	Pain in the extremities	Seizures	Dark urine	Skin lesions	Blood pressure (mmHg)	Pulse rate/min	Watson–Swartz test	Urinary urobilinogen	Urinary urobilinogen/ Serum TBIL ratio	Gene mutation
1	Female	20	Menstruation	Yes	No	No	No	No	149/87	112	Positive	200	10.52	N/A
2	Male	24	Alcohol	Yes	Yes	Yes	No	No	107/62	101	Positive	70	4.96	N/A
3	Female	37	Pregnancy	Yes	No	No	No	No	146/94	86	Positive	70	7.00	Yes (HMBS)
4	Female	28	Pregnancy	Yes	Yes	Yes	No	No	137/80	104	Positive	140	7.36	N/A
5	Female	32	Menstruation	Yes	No	Yes	No	No	111/87	116	Positive	70	5.30	N/A
6	Female	19	Unknown	Yes	Yes	No	Yes	No	144/113	123	N/A	N/A	N/A	Yes (HMBS)
7	Female	23	Menstruation	Yes	Yes	No	No	No	169/120	112	Positive	140	7.00	N/A
8	Female	35	Menstruation	Yes	No	No	Yes	No	161/89	86	Positive	70	5.93	N/A
9	Female	29	Unknown	Yes	No	No	No	No	97/68	122	Positive	70	7.07	N/A
10	Female	27	Unknown	Yes	No	No	No	No	150/78	78	Positive	35	3.88	N/A
11	Female	18	Unknown	Yes	Yes	No	No	No	129/72	123	N/A	200	4.76	Yes (HMBS)
12	Female	25	Menstruation	Yes	Yes	Yes	No	No	129/92	81	Positive	140	4.37	Yes (HMBS)

HMBS: Hydroxymethylbilane Synthase.

Interestingly, half of the AHP patients were first diagnosed by endocrinologist during a consultation for hyponatremia. For the other six cases, four were diagnosed by neurologists, and two were diagnosed by gastroenterologist and surgeon. During their previous visits, these patients had been misdiagnosed as intestinal obstruction (83.3%), undifferentiated abdominal pain (75.0%), appendicitis (8.3%), and seizure (16.7%). Appendectomy was performed in one patient at the primary hospital and seizures happened after the surgery.

### Elevated urinary urobilinogen was found in AHP patients

One hundred patients with abdominal pain caused by other diseases were enrolled in the control groups. During the acute attack, the AHP patients showed significant lower hemoglobin, serum sodium, serum chlorine, and “falsely” higher urinary urobilinogen levels compared with control groups. In most of the AHP patients, the serum total bilirubin level was normal, and no significant difference was found when compared with the control groups (Table 2).

**Table 2:** Comparison of laboratory results between acute hepatic porphyria patients and patients with abdominal pain due to other causes.

	Cholecystitis and gallstones (N = 25)	Intestinal obstruction (N = 25)	Pancreatitis (N = 25)	Appendicitis (N = 25)	Acute hepatic porphyria (N = 12)
Male/female ratio	13:12	10:15	13:12	10:15	1:11
Age	67.00 (60.50,69.00)**	52.96 ± 16.04**	52.48 ± 15.48**	42.80 ± 17.97**	27.73 ± 6.77
Hemoglobin (range 130–175 g/L)	133.64 ± 16.08*	133.16 ± 18.25*	140.29 ± 21.35**	137.04 ± 20.37*	107.75. ± 13.79
ALT (range 21–72 U/L)	37.00(20.50,137.00)	22.50 (20.00, 27.75)**	63.50 (33.50,233.50)	25.00 (20.00, 42.00)**	41.50 (32.50,83.00)
AST (range 17–59 U/L)	28.00 (23.50,150.00)	26.00 (21.00, 34.00)*	47.00 (32.00,199.00)	23.00 (19.50, 33.00)*	43.00 (29.50,106.60)
Serum TBIL (range 3–22 μmol/l)	16.00 (10.50,35.50)	14.00 (11.00, 20.50)	20.00 (12.50,32.00)	16.00 (10.00, 22.00)	13.00 (10.90,19.50)
Serum DBIL (range 0–5 μmol/l)	0.00 (0.00, 0.00)	0.00	0 (0.00, 0.00)	0.00	0.00
Serum IBIL (range 0–19 μmol/l)	9.00 (7.00, 18.00)	11.00 (7.00,16.00)	13.00(8.50, 23.50)	11.00 (7.00, 16.00)	11.38 ± 7.34
Serum amylase (range 30–110 U/L)	69.50 (57.25, 83.00)	72.00 (59.00, 72.00)	531.00 (238.00,1021.50)**	75.50 ± 34.00	65.25 ± 27.04
Serum sodium (range 137–145 mmol/l)	136.43 ± 3.37**	135.46 ± 3.71**	136.25 ± 3.08**	137.42 ± 4.34**	128.17 ± 8.93
Urinary urobilinogen	0.00 (0.00, 0.00)**	0.00 (0.00, 0.00)**	0.00 (0.00, 0.00)**	0.00 (0.00, 0.00)**	109.55 ± 57.03
Urinary urobilinogen/Serum TBIL ratio	0.00 (0.00, 0.00)**	0.00 (0.00, 0.00)**	0.00(0.00, 0.00)**	0.00 (0.00, 0.00)**	6.19 ± 1.88

*ALT* alanine transaminase; *AST* aspartate transaminase; *DBIL* direct bilirubin; *IBIL* indirect bilirubin; *TBIL* total bilirubin.

*P < 0.05, **P < 0.01.

### Elevated urinary urobilinogen in AHP patients was a false-positive result caused by urinary PBG

**Figure 1:**
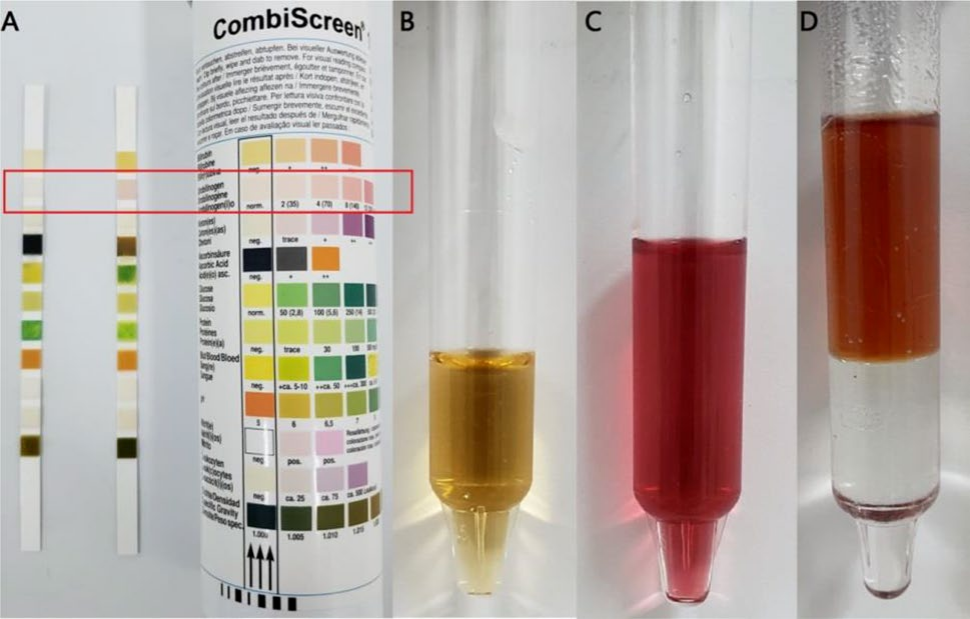
Elevated levels of urinary urobilinogen in acute hepatic porphyria patients were false-positive results caused by urinary PBG. (**A**) A positive urinary urobilinogen result reported by the dipstick (the right dipstick in the red box). (**B**) Fresh urine sample of AHP patient. (**C**) Urine sample turned red after adding an equal volume of Ehrlich’s reagent. (**D**) Red color in the aqueous phase of the test tube after adding chloroform illustrated the existence of the PBG-Ehrlich compound, whereas no color in the chloroform phase excluded the existence of the urobilinogen-Ehrlich complex.

PBG, which is greatly increased during an acute attack of AIP, VP and HCP, can also react with Ehrlich's reagent. So, we suspected that the high urinary urobilinogen level without elevated serum total bilirubin in AHP patients was a false-positive result caused by the urinary PBG. Watson–Schwartz test was used to confirm this speculation (Fig. 1). After an equal volume of Ehrlich’s reagent was added, the urine sample of AHP patient turned red and proved the existence of PBG-Ehrlich compound or urobilinogen-Ehrlich complex (Fig. 1C). And then chloroform was added to the solution to distinguish these two substances. Red color in the aqueous phase of the test tube illustrated the existence of the PBG-Ehrlich compound, whereas no color in the chloroform phase excluded the existence of the urobilinogen-Ehrlich complex (Fig. 1D).

### Performance of urinary urobilinogen/serum total bilirubin ratio as an indicator for AHP

**Figure 2:**
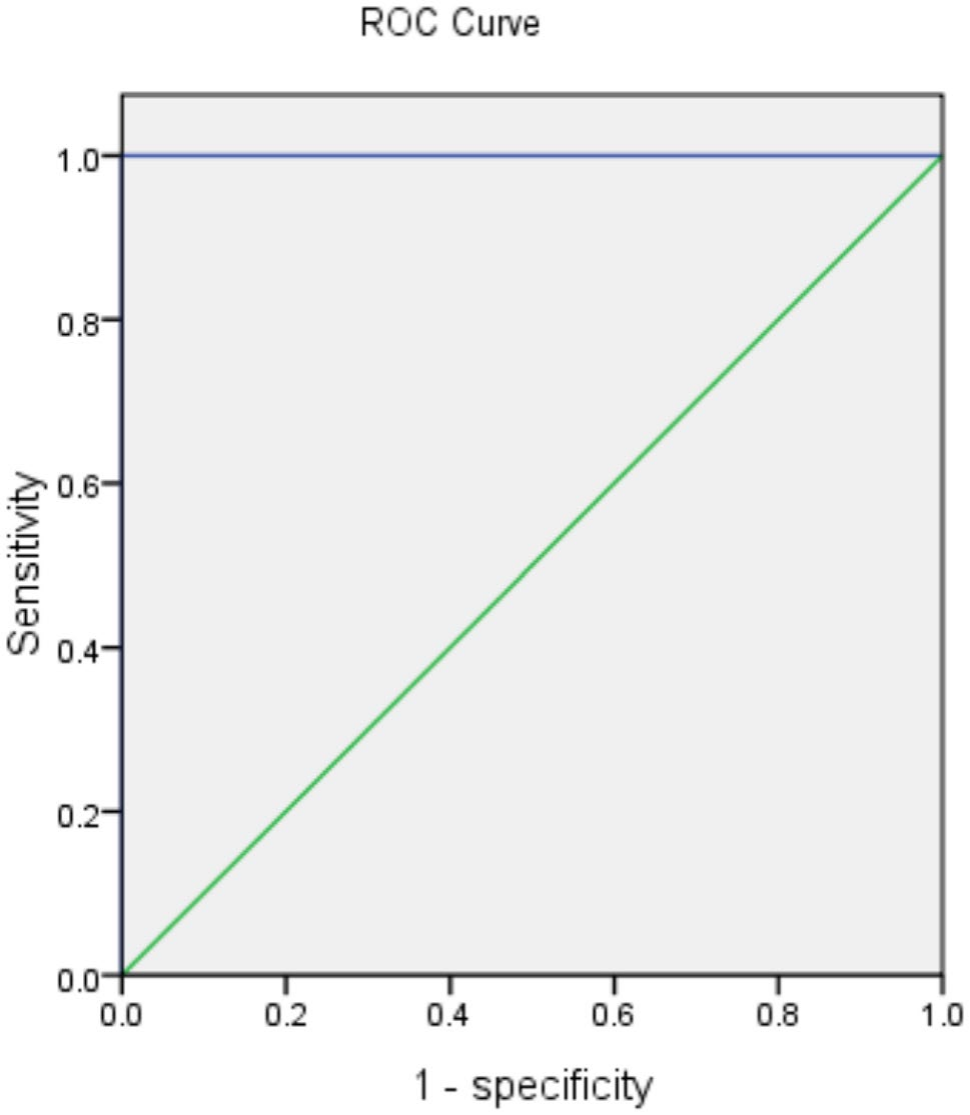
Diagnostic performance of urinary urobilinogen/serum total bilirubin ratio for acute hepatic porphyria. ROC curves were plotted using data from the acute hepatic porphyria patients and abdominal pain patients of other causes to assess the performance characteristics of urinary urobilinogen/serum total bilirubin ratio for acute hepatic porphyria. AUC was 1.000 with a 95% CI of 1.00–1.00. Cutoff point = 3.22.

Because urinary urobilinogen was a false-positive result caused by urinary PBG, we used serum total bilirubin, an upstream substance of urinary urobilinogen synthesis, for calibration. A remarkable increase in urinary urobilinogen/serum total bilirubin ratio in AHP patients (6.19 ± 1.88 vs. 0.00 ± 0.0, *P* < 0.01) was observed when compared with that in the control groups (Table 2). The performance of urinary urobilinogen/serum total bilirubin ratio as an indicator for AIP, HCP and VP was assessed by generating a ROC curve (Fig. 2). Since one patient did not undergo the urinary examination, only data from eleven AHP patients were used for the ROC analysis. With the 100 samples from abdominal pain patients of other causes as the controls, the area under the ROC curve of urinary urobilinogen/serum total bilirubin ratio for AHP was 1.000 (95% CI 1.000–1.000, *P* < 0.01). When using the maximum paired sensitivity and specificity values from the ROC curve, we determined a cutoff value of 3.22 for the urinary urobilinogen/serum total bilirubin ratio in indicating AIP, HCP and VP from abdominal pain patients. The sensitivity, specificity, PPV, and NPV of the urinary urobilinogen/serum total bilirubin ratio to identify patients with AHP were 100%, 100%, 100%, and 100%, respectively.

## Discussion

In this retrospective study, we discovered that the urinary urobilinogen was greatly increased in the AHP patients when compared with the control groups. And we proved the elevation was a false-positive result caused by urinary PBG. Calibration with serum total bilirubin, an upstream substance of urinary urobilinogen synthesis, could help to distinguish the false-positive from true-positive result, and provide for more frequent diagnosis of AHP even when that diagnosis is not suspected.

Common presentations of AHP include abdominal pain (85–95%), constipation (48–84%), extremity pain (50–52%), nausea and vomiting (43–88%), tachycardia (28–80%), and hypertension (36–54%)^3^. Bullous skin lesions may be present during an attack of VP or HCP^14^. In severe cases, seizures, hallucination and respiratory distress might also present and patients could require intensive care^15^. In our study, 100% of patients complained of abdominal pain, which was much higher than previous reported. This might be due to a potential missed diagnosis of AHP patients with other symptoms caused by the poor awareness of physicians in our hospital.

The key issue in AHP management is to suspect the diagnosis^16^. In our study, we found that 50% of the patients were diagnosed by endocrinologist, which was rare in the published articles. This finding highlights the importance of experienced physicians for AHP diagnosis. However, as a rare disease, awareness of AHP in China is limited(11) and the misdiagnosis rate of AHP was 70% in a tertiary hospital in China^13^. Thus, finding an indicator during routine examinations is needed to help physicians notice the possibility of AHP.

In the present study, we reported that the AHP patients showed a high level of apparent urinary urobilinogen for the first time. However, the serum bilirubin level was normal or only slightly elevated, consistent with the literature^10,13,15,17,18^. Urobilinogen is a product of bilirubin metabolism by anaerobic bacteria in the intestine. The majority of the reabsorbed urobilinogen is taken up by the liver and then re-excreted into bile, while a small amount is excreted in the urine^19^. In certain diseases, such as hemolytic anemia, hepatic jaundice, and biliary disease, the serum bilirubin level is greatly elevated and leads to the excessive production of urobilinogen^20,21^. However, in people with normal serum bilirubin, the urinary urobilinogen is negative since the amount of urinary urobilinogen is too low to be detected. So, we suspected the elevated urinary urobilinogen in AHP patients was a false-positive result caused by PBG and proved it by using the Watson–Schwartz test.

Both PBG and urobilinogen can react with Ehrlich's reagent and form a red condensation product in the first step of Watson–Schwartz test^22^. As such, in the second step of the test, chloroform is added to the solution to distinguish the PBG-Ehrlich compound from the urobilinogen-Ehrlich complex. However, it is impossible to use chloroform to distinguish these substances when the reaction happened on the pad of the dipstick. In that case, calibration by serum bilirubin (urinary urobilinogen/serum total bilirubin ratio) is a good way to help the doctors to identify the false-positive urinary urobilinogen and further suspect the diagnosis of AHP. Via ROC curve analysis, we found that the cutoff point of this ratio for AHP diagnosis was 3.22. So, in patients with typical clinical symptoms such as abdominal pain, the AHP diagnosis should be considered and further investigation of urinary PBG and ALA should be carried out when the urinary urobilinogen was positive, especially when the urinary urobilinogen/serum total bilirubin ratio was above 3.22. It is worth noting that sulfonamides, *p*-aminosalicylic acid, and drugs containing Azo dyes (nitrofurantoin, riboflavin, methyldopa) could also react with Ehrlich’s reagents^23^, so a detailed history taking is necessary.

The potential clinical use of the urinary urobilinogen suggests the importance of urinalysis in the diagnosis of urinary PBG-elevated AHP. By far, urinalysis is routinely carried out in inpatients in China, greatly promised the feasibility of this marker for AHP screening. However, urinalysis is often overlooked both by patients and physicians in the emergency department and clinic. In fact, dark urine is very common in AHP patients and sometimes becomes the first clue for AHP patients with seizures in the intensive care unit^5,24^. However, all the urine specimens showed amber color during urinalysis, but only two patients mentioned a change of urine color in their complaints in the present study. In addition, a menstrual period is a common predisposing factor of an acute attack, but urinalysis is often avoided both by the physicians and female patients during their periods. Therefore, we strongly suggest that all patients with abdominal pain undergo urinalysis and the serum total bilirubin which are both routine and cost-effective examinations.

Our study has several limitations. Since the urinary ALA examination were not available in our hospital, it is difficult to identify the accurate subtype of AHP. Meanwhile, neither the urinary urobilinogen nor PBG is quantitative detection, so we could not analyze the relation between these two results. Moreover, since AHP is a rare disease, we only enrolled 12 patients from one single center in this study. Due to the limited sample size and semiquantitative result of urinary urobilinogen, there might be variations in the cut-off point. Future multi-center studies with larger sample sizes are needed.

## Conclusions

In patients with abdominal pain, the urinary urobilinogen/serum total bilirubin ratio can be used as an indicator for AIP, HCP and VP. With a cutoff point of 3.22, this ratio had a specificity and sensitivity of 100% and 100%, respectively. This finding may greatly improve the diagnosis of AIP, HCP and VP. When the urinary urobilinogen is “falsely” positive during the urinalysis when detected by strips impregnated with Ehrlich reagent, especially when the ratio is higher than 3.22, further investigation of urinary PBG, ALA or a gene test is recommended.

## Data Availability

The datasets used and analyzed in the present study are available from the corresponding author on reasonable request.

## Abbreviations

AHP: Acute hepatic porphyria
PBG: Porphobilinogen
ALA: 5-Aminolevulinic acid
RBC: Red blood cell
ALT: Alanine transaminase
AST: Aspartate transaminase
TBIL: Total bilirubin
DBIL: Direct bilirubin
IBIL: Indirect bilirubin
